# Implementing Digital Trainings within Medical Rehabilitations: Improvement of Mental Health and Synergetic Outcomes with Healthcare Service

**DOI:** 10.3390/ijerph18178936

**Published:** 2021-08-25

**Authors:** Franziska Maria Keller, Alina Dahmen, Christina Derksen, Lukas Kötting, Sonia Lippke

**Affiliations:** 1 Department of Psychology and Methods, Jacobs University Bremen, 28759 Bremen, Germany; f.keller@jacobs-university.de (F.M.K.); c.derksen@jacobs-university.de (C.D.); l.koetting@jacobs-university.de (L.K.); 2 Dr. Becker Klinikgruppe, 50968 Cologne, Germany; adahmen@dbkg.de

**Keywords:** mental health, psychosomatic rehabilitation, internet-delivered digital trainings, multidisciplinary and interdisciplinary interventions

## Abstract

The need for new technologies in healthcare services has been stressed. However, little is known about the effectiveness of digital interventions integrated in psychosomatic rehabilitation processes. Data from 724 patients from psychosomatic rehabilitation clinics were analyzed with regard to the effectiveness of digital trainings indicated by a change in symptoms related to depression, anxiety, stress, and loneliness from pre– to post–rehabilitation. Rehabilitation satisfaction was examined in association with reaching rehabilitation goals and satisfaction with communication. A mixed repeated measures analyses of covariance, analyses of covariance, and hierarchical stepwise regression analyses were performed. Results indicated a superior effectiveness for the intervention group receiving all offered digital treatments in addition to the regular face-to-face rehabilitation program with regard to symptoms of depression (F (2674) = 3.93, *p* < 0.05, η_p_^2^ = 0.01), anxiety (F (2678) = 3.68, *p* < 0.05, η_p_^2^ = 0.01) post-rehabilitation, with large effect sizes for both depression (d = 1.28) and anxiety (d = 1.08). In addition, rehabilitation satisfaction was positively associated with reaching rehabilitation goals and perceived communication with healthcare workers. Digital interventions appeared effective in supporting mental health of psychosomatic rehabilitation patients’ post-rehabilitation. These findings support the inclusion of multidisciplinary and interdisciplinary digital and face-to-face treatment programs and call for more implementations of new technologies in a context of complexity to improve health and healthcare service.

## 1. Introduction

### 1.1. Mental Health and the COVID-19 Pandemic

The effects of the coronavirus disease 2019 (COVID-19) on individuals’ health, especially on mental health and perceived well-being, are likely to be profound and long-lasting [1]. Not only has the COVID-19 pandemic lead to rapid changes in human interaction, hygiene behavior, communication behavior, and self-care, but it has also led to increased feelings of uncertainty, distress, and social isolation resulting in stress reactions, symptoms of depression and anxiety, and general fear of the virus [2]. Several studies have pointed out that elevated rates of depression, anxiety, and stress, as well as post-traumatic stress were associated with the COVID-19 pandemic [3]. Therefore, in case of a prolongation of restriction measures, individuals, especially those who are already susceptible to a mental health disorder, may develop serious mental health issues [4,5].

For individuals with a pre–existing mental health disorder, the lockdown measures have shown to be major stress factors and are associated with a deterioration of their mental health status due to changes in daily routine and social rhythms [5], reduced access to support services, earlier discharge from psychiatric units, or discontinuation of psychotherapy treatments [6,7,8].

To partially compensate for reduced access to support systems and discontinuation of psychotherapy, therapists have been more prone to offer digital psychotherapy sessions in addition to face-to-face sessions to guarantee the continuation of treatment as well as to protect and support the mental health of patients. The idea of blended psychotherapy as a combination of online treatment with face-to-face psychotherapy is rather a new research field and has received more attention during the COVID-19 pandemic [9,10,11,12]. Blended psychotherapy has shown to provide many advantages over face-to-face psychotherapy as it supports bridging distances between residence and treatment placement, flexibility, as well as increased patient empowerment [13,14].

However, even though a few studies have examined the effectiveness of blended psychotherapy in outpatient settings [15], the effectiveness of integrating digital interventions in form of a blended psychotherapy concept into medical, psychosomatic rehabilitation treatment programs has yet to be evaluated.

### 1.2. Therapy for Medical, Psychosomatic Rehabilitation Patients

The overarching aim of the rehabilitation system in Germany is to reintegrate and support social participation of patients but not to curate disorders. Patients admitted to rehabilitation clinics in Germany are generally treated on the basis of the biopsychosocial model [16]. This is in contrast to patients with severe mental health disorders who are typically seen by a psychiatrist and are potentially being admitted to a psychiatric hospital and treated by an interdisciplinary and multidisciplinary team according to the German national regulations and guidelines. Hence, rehabilitation is usually an in-patient program providing psychoeducation, psychotherapy in from of individual and group therapy, physical therapy, and occupational therapy, as well as trainings of skills relevant for the reintegration and return to work (RTW) [17]. One of the skills necessary for reintegration and social participation is effective communication. Therefore, during psychotherapy sessions, patients are informed about, encouraged, and supported to improve their communication skills in order to obtain and maintain a healthy mental well-being.

Research has shown that blended therapy can be well integrated in the preparatory process before a rehabilitation stay [18], during the rehabilitation process at the rehabilitation clinics itself [19], and for aftercare and stabilization [20]. First attempts to provide patients with digital support after rehabilitation have already been made by the Curriculum Hannover Online [21] and the internet– and mobile–based intervention (IMI) DE-RENA [22]. Although digital trainings are usually accepted as suitable in the context of healthcare and rehabilitation (particularly to buffer negative consequences of the COVID-19 pandemic and ensure treatment options), methods and techniques need to be carefully planned and implemented. A non-suitable training might affect rehabilitants’ health and well-being negatively since no immediate support is available in a digital setting if trainings pose a strain on individuals with mental health symptoms. Additionally, digital interventions might negatively affect rehabilitants’ treatment motivation if contents and mode of delivery are not suitable, thus inhibiting effective inpatient rehabilitation. However, so far, no study has attempted to evaluate digital trainings offered before or during medical, psychosomatic rehabilitation.

### 1.3. Importance of Communication in Medical, Psychosomatic Rehabilitation Treatment Programs

Communication is the central element of psychotherapy and thus a central element of the psychosomatic rehabilitation process. Based on a patient’s verbal and nonverbal communication, psychotherapists are able to foster a diagnostic-therapeutic alliance with the patient [23,24,25]. Further, patients are encouraged by trainings, such as elements of the social competence training, to learn and apply effective communication strategies. Additionally, the therapist is encouraged to promote effective communication strategies tailored to the individual patient in order to understand maladaptive behaviors and to support the patient with treatment options and coping skills [26]. It has been shown that effective communication skills with patients throughout the therapeutic process (i.e., through transparency, goal-value clarification, or through an empathic approach) foster and encourage therapy motivation as well as motivation to change. One strategy that has been proven effective with regard to the treatment of depression and anxiety is communication-focused therapy (CFT; [27,28]). CFT assumes that changes as part of the therapeutic process (i.e., changes in learning processes, as well as acceptance and behavioral adaptions) are determined by results of effective communication processes between a patient and a therapist. Therefore, to encourage a therapeutic alliance in order to provoke therapeutic changes, therapists are encouraged to improve their own communication skills relevant for the healthcare context.

An example of communication skills required in the healthcare context was proposed by Rider and Keefer [29]. In the study, the authors highlighted the importance to communicate effectively with patients by focusing on interpersonal relations. Hence, healthcare professionals are encouraged to communicate clearly and accurately and to provide the patient with sufficient information by also acknowledging the patient’s individual situation. However, with regard to the setting of a medical, psychosomatic rehabilitation treatment, the exact association between perceived effectiveness of communication from the patients’ perspective and rehabilitation effectiveness as well as satisfaction has so far not been evaluated. 

### 1.4. Compensatory Carry-Over Action Model

One theoretical model that describes the relationship between mental well-being and factors associated with well-being is the Compensatory Carry-Over Action Model (CCAM [30]). The CCAM describes how health outcomes, such as a decrease in symptoms of depression and anxiety or perceived loneliness and stress, result from different health-related behaviors such as participation in digital trainings, adaptions of communication behavior, and how they change also as a result of one another. In addition, the CCAM assumes that relevant, higher-order goals such as workability and participation may be achieved by implementing goals for individual health-related behaviors through the use of action plans. Important for the transfer between the individual behaviors (i.e., participation in digital trainings or improved communication competencies and reduced mental health symptoms) are personal psychological resources [30]. 

The psychiatrist or therapist fosters an early diagnostic-therapeutic alliance with the patient. S/he integrates information obtained through both the patient’s verbal and nonverbal communication and his or her own countertransference [23,24]. Therefore, the CCAM provides the theoretical basis to explain how adaptions to the rehabilitation process (i.e., by offering digital trainings, supporting goal attainment, or improving communication competencies of therapists) are associated with a change in symptoms of depression, anxiety, perceived loneliness, and stress from pre– to post–rehabilitation.

### 1.5. Goal of the Study

The goal of the current study was to test the effectiveness of digital trainings provided to rehabilitation patients before and during their medical rehabilitation stay. In addition, as communication is a central element of (psycho)therapy, the present study aimed to evaluate the interrelation of communication with rehabilitation satisfaction and consequently with perceived rehabilitation success.

With these research aims in mind and on basis of the theoretical background of the CCAM and previous findings, the following hypotheses were formulated. (1) Symptoms of depression, anxiety, perceived stress, and loneliness will decrease from pre– to post–rehabilitation. Additionally, we expected that (2) the intervention group who received all digital trainings will have a more substantial decrease of symptoms with regard to depression, anxiety, perceived stress, and loneliness. Further, we assumed that (3) the patients from the intervention group receiving all digital trainings will indicate a higher perceived rehabilitation success. We also predicted that (4) patients who perceived greater satisfaction with communication will be more satisfied with their rehabilitation process and will more likely indicate that they have achieved their rehabilitation goals.

## 2. Materials and Methods

### 2.1. Study Design

The present study was conducted at four psychosomatic rehabilitation clinics from the Dr. Becker clinic group in a longitudinal manner. Participants recruited to this study received regular treatment programs with regard to psychological and physical interventions. Those treatment programs included among others individual and group psychotherapy, physiotherapy, and occupational therapy.

### 2.2. Recruitment and Data Collection

Participants were recruited through the four participating clinics from the Dr. Becker clinic group. Before participation, patients were informed about the study in writing on the rehabilitation clinic group’s online portal. Thereby, it was guaranteed that only patients who had access to the digital portal with an individualized participant code could participate. Patients were invited to take part in a survey administered via the survey platform Unipark. Before participating in the survey, patients were asked to read the participation information and were asked to give informed consent. All data collected as part of this study were pseudonymized. Participants were not offered any form of compensation for participating in the study. The survey at the four psychosomatic clinics was administered between July 2020 and June 2021. Data collection was longitudinal with two measurement time points. Patients were invited to participate from six weeks before until the first day of rehabilitation (T1) as well as after their rehabilitation stay (T2). Participation after rehabilitation was possible for a maximum of 12 weeks post-rehabilitation. Reminders were sent out to the participants for the T2 survey after 1, 4, and 11 weeks post–rehabilitation. Ethical approval for the online survey concerning psychosomatic rehabilitation patients was given by the Ethics Committee at Jacobs University Bremen (protocol code 2020_09 and date of approval: 25 June 2020). The current study was conducted as part of the project “Anhand-COVID19-Offer to achieve treatment and rehabilitation goals in compliance with hygiene and social-distancing rules” (ClinicalTrials.gov Identifier: NCT04453475), which is supported by the Dr. Becker clinic group.

### 2.3. Participants

In total, *n* = 1279 patients participated in the online survey at timepoint 1 (before rehabilitation stay). A total of 555 patients dropped out after the baseline assessment, leaving 724 participants who completed the survey at measurement timepoint 1 pre–rehabilitation as well as the survey at measurement timepoint 2 post–rehabilitation.

The most common three diagnoses that patients received, according to the International Classification of Disease-10 (ICD–10) manual, were as follows: a major depressive disorder, recurrent, moderate (F33.1) with *n* = 193 (26.7%); an adjustment disorder (F43.2) with *n* = 159 (22.0%); and a major depressive disorder, single episode, moderate (F32.1) with *n* = 93 (12.8%). Patients’ ages ranged from 18 to above 60 years. Within the sample, 466 (64.4%) patients were female. Additionally, 155 (21.7%) patients had a secondary school diploma, 106 (14.8%) patients had a high school diploma, 319 (44.6%) patients had completed vocational training, and 135 (18.9%) patients indicated having a university degree.

### 2.4. Interventions

As part of the incoming process and prior to the begin of the treatment stay, participants were asked to participate in a digital training on rehabilitation goals presented to patients in a digital PowerPoint presentation without face-to-face elements. Participation was on a voluntary basis. The digital training on rehabilitation goals could be accessed from home with a computer, laptop, tablet, or smartphone. This training was designed as a combination between psychoeducation and practical elements. Patients were educated on the importance of formulating goals and plans as well as on how to formulate those. After the educative element, participants were instructed to formulate their own plans for their rehabilitation treatment process. Further interactive tools such as digital exercise booklets supporting goal and plan formulation were provided to patients online. Patients were encouraged to make use of the supporting material after the training.

As part of the rehabilitation process, participants diagnosed with a major depression were required to take part in the digital group training for depression. The digital group training was based on cognitive behavioral therapy (CBT) guidelines with evidence-based components of computerized cognitive behavioral therapy (eCBT) and internet-delivered cognitive behavior therapy (iCBT) interventions [31,32,33]. The group therapy for depression was conducted in a flipped classroom manner with a combination of digital and face-to-face components. The digital group therapy for depression was divided into six therapy sessions. Each session lasted for about 50 min. The 50 min sessions were divided into a 5 min digital training followed by a 45 min analog group session. Contents discussed during the group sessions included psychoeducation on the symptoms of and coping mechanisms for depression, underlying models, as well as different available treatments such as drug therapy and ambulatory or stationary psychotherapy.

The informative digital training on legal rights for (severely) disabled was offered to all patients once during their rehabilitation stay in the form of a group session. Participation was mandatory irrespective of the ICD-10 diagnosis. The training consisted of a 20 min informative video and a subsequent 25 min face-to-face group session in which in-depth questions were discussed in accordance with the flipped classroom manner. Contents of the video and the group discussion included aspects of the law on severe disabilities, requirements for obtaining a degree of disability, and its consequences on everyday life.

Hence, the study design was set up as follows: participants allocated to the control group received the care-as-usual rehabilitation program. Patients allocated to intervention group 1 (IG1) took part in the digital training on rehabilitation goals prior to the rehabilitation stay in addition to the care-as-usual rehabilitation program. As part of intervention group 2 (IG2), patients took part in the digital training on rehabilitation goals prior to the rehabilitation stay, the digital group therapy on depression, and the digital training on legal rights for (severely) disabled in addition to the regular care-as-usual rehabilitation treatment.

### 2.5. Instruments

#### 2.5.1. Depressive Symptoms and Symptoms of Anxiety

To measure symptoms of depression and anxiety, the Patient Health Questionnaire-4 (PHQ-4) was used. The questionnaire was not used as a diagnostic tool as part of this study, but rather used as a measure of symptom intensity. The PHQ-4 is a composite measure with four items of the PHQ-2 [34] and the GAD-2 [35]. All four items are measured on a 4-point Likert scale from 0 (‘not at all’) to 3 (‘nearly every day’). A scale sum score of ≥3 for both the PHQ-2 (T1 Spearman’s rho = 0.70; T2 Spearman’s rho = 0.71) and the GAD-2 (T1 Spearman’s rho = 0.64; T2 Spearman’s rho = 0.67) depicts the cut-off value between the normal range and a probable case of depression and anxiety [36,37].

#### 2.5.2. Perceived Stress

As a measure of stress, the Perceived Stress Scale (PSS; [38]) was used. The PSS is a globally used self-report scale measuring perceived stress. With regard to the current study, perceived stress was measured by the short four-item version of the PSS scale (PSS-4; [39]). The PSS-4 assesses perceived stress on a 5-point Likert scale from 0 (‘never’) to 4 (‘very often’) with a Cronbach’s alpha at T1 of 0.71 and at T2 of 0.85.

#### 2.5.3. Loneliness

Perceived loneliness was assessed by means of two items: ‘How often do you feel lonely?’ stemming from the Center for Epidemiologic Studies–Depression (CES–D) Scale [40] and ‘How often do you feel unhappy to be alone?’ from the UCLA Loneliness Scale [41] (T1 Spearman’s rho = 0.81, T1 Spearman’s rho = 0.81, respectively). Both items were measured on a 4-point Likert scale from 1 (‘not at all’) to 4 (‘almost every day’).

#### 2.5.4. Rehabilitation Goals

Before and after rehabilitation, patients were asked to indicate whether they aimed to achieve eight possible rehabilitation goals on a scale from 1 (‘not at all’) to 4 (‘completely’) with a Cronbach’s alpha at T1 of 0.65 and at T2 of 0.89. Examples of possible rehabilitation goals included the reduction of mental health symptoms, an improvement of stress coping capabilities, improvement of cognitive abilities, ability to relax and rest, or the improvement of/return to past earning capacities. The items assessing rehabilitation goals were developed based on the provided content and the outcome aims of the digital trainings provided before and during the rehabilitation treatment.

#### 2.5.5. Perceived Communication

Perceived communication between rehabilitation patients and healthcare professionals (i.e., psychotherapists, occupational therapists, doctors, nurses, or other healthcare staff) was examined from the perspective of rehabilitation patients through six items developed based Rider and Keefer’s interpersonal communication competencies with a Cronbach’s alpha of 0.88 [29].

#### 2.5.6. Satisfaction with Rehabilitation

Post rehabilitation, patients were asked to indicate their satisfaction with medical rehabilitation with one item on a 6-point Likert scale from 1 (‘very dissatisfied’) to 6 (‘very satisfied’).

#### 2.5.7. Statistical Analyses

For all analyses, SPSS Version 27 was used (IBM Corp., Armonk, NY, USA). The data were analyzed using 724 patients who were either allocated to the control group or to the intervention groups (IG1 to IG2). A randomization check was performed to confirm successful allocation to groups. Hence, the different groups (control group and two intervention groups) were compared for age, gender, educational status, symptoms of depression and anxiety, perceived stress, and perceived loneliness before rehabilitation. Therefore, a one-way ANOVA was used for continuous variables. Chi-squared tests were used for nominal variables. According to Tabachnick and Fidell [42], it has been suggested that, in case of a significant difference, correlations between the significant variable and the dependent variable should be computed to assess whether the significant variable should be included as a covariate in our analyses.

As the amount of missing data was below 5% for all items, no imputation of missing data was performed. Patients with missing data on the social-cognitive variables (i.e., age or gender) were included for further analyses if they had at least one non-missing data point under the assumption of missing data (completely) at random.

To evaluate significant changes in the symptom intensity with regard to depression, anxiety, stress, and loneliness, a 2 × 3 linear mixed-model repeated measures analysis of covariances (MMRM ANCOVA) was performed. In order to explain a significant time x intervention group interaction effect, the mean difference scores for the two time points were computed (before rehabilitation to after rehabilitation). Based on the mean difference scores, a series of analyses of covariances were performed to identify differences between the interventions.

To determine the effect sizes of all measurements, partial eta squared and Cohen’s d values were computed. Based on the recommendation by Field [43], partial eta squared values of 0.01, 0.06, and 0.14 represent weak, moderate, and strong effects, respectively [44,45]. Cohen’s d values of 0.20, 0.50, and 0.80 represent small, medium, and large effect sizes, respectively [44]. 

Further, we performed several multivariate analyses of covariance (MANCOVA) to evaluate which treatment group was more likely to reach the proposed rehabilitation goals. Additionally, we investigated the association between the estimation of achieving rehabilitation goals and overall satisfaction with rehabilitation treatment by means of a stepwise hierarchical linear regression.

## 3. Results

### 3.1. Randomization Check

With regard to the current study, n = 55 (7.6%) patients did not participate in any of the three digital trainings and were thus defined as the control group. Overall, 570 (78.7%) patients participated in the digital trainings on rehabilitation goals (intervention group 1—IG1), 80 (11.0%) patients participated in all three digital trainings (intervention group 2—IG2), and 19 patients (2.6%) were excluded from the analyses.

There were no significant differences for gender (X^2^ (2, n = 701) = 1.60, *p* = 0.45), for age (X^2^ (8, n = 703) = 11.84, *p* = 0.16), and for educational level (X^2^ (6, n = 696) = 2.80, *p* = 0.83). In addition, there were no significant differences for symptoms of depression (F (2695) = 0.78, *p* = 0.46, η_p_^2^ = 0.01), for symptoms of anxiety (F (2698) = 1.15, *p* = 0.32, η_p_^2^ = 0.01), as well as for perceived stress (F (2695) = 1.61, *p* = 0.20, η_p_^2^ = 0.01) and perceived loneliness (F (2686) = 1.22, *p* = 0.30, η_p_^2^ = 0.01).

### 3.2. MMRM ANCOVA from before Rehabilitation Treatment to after Rehabilitation Treatment

Results indicated a significant main effect across time, controlling for age and gender for symptoms of depression (F (1674) = 13.34, *p* < 0.01, η_p_^2^ = 0.02), symptoms of anxiety (F (1678) = 6.80, *p* < 0.01, η_p_^2^ = 0.01), and perceived stress (F (1672) = 17.63, *p* < 0.01, η_p_^2^ = 0.03), as well as for perceived loneliness (F (1662) = 4.00, *p* < 0.05, η_p_^2^ = 0.01).

A significant interaction between time x intervention, controlling for age, gender, and intervention group, emerged for symptoms of depression (F (2674) = 3.93, *p* < 0.05, η_p_^2^ = 0.01) and symptoms of anxiety (F (2678) = 3.68, *p* < 0.05, η_p_^2^ = 0.01). However, no significant interaction effect was found for perceived stress (F (2672) = 1.80, *p* = 0.17, η_p_^2^ = 0.01) as well as for perceived loneliness (F (2662) = 2.69, *p* = 0.07, η_p_^2^ = 0.01).

No significant main effect for intervention, controlling for age and gender, was found for all four outcome domains: depression (F (2774) = 0.58, *p* = 0.56, η_p_^2^ = 0.01), anxiety (F (2678) = 0.42, *p* = 0.66, η_p_^2^ = 0.01), perceived stress (F (2672) = 0.832, *p* = 0.44, η_p_^2^ = 0.01), and loneliness (F (2662) = 1.43, *p* = 0.24, η_p_^2^ = 0.01). Reported effect sizes for the main effects of time and intervention as well as for the interaction effect of time x intervention were small for all outcome domains.

### 3.3. Changes in Mental Health Symptoms with Regard to the Intervention Group from before Rehabilitation Treatment and after Rehabilitation Treatment

**Figure 1:**
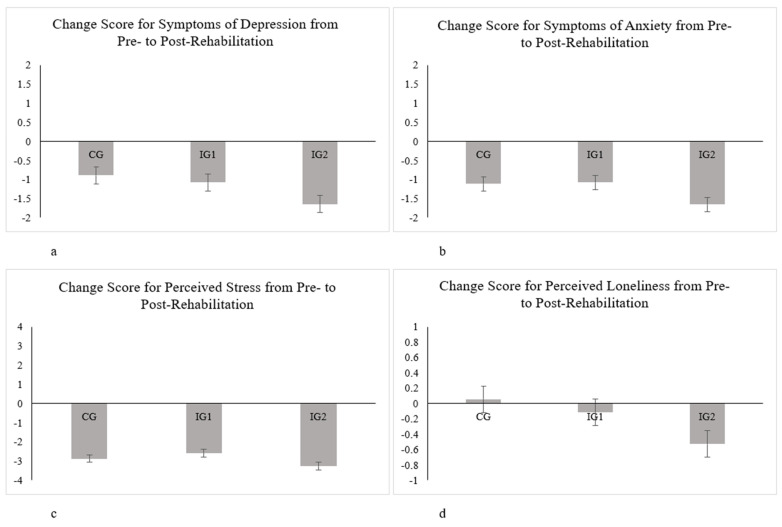
Estimated marginal means for symptoms of depression (**a**), symptoms of anxiety (**b**), perceived stress (**c**), and perceived loneliness (**d**). Error bars are represented by standard errors of the mean. Higher negative scores represent a greater reported symptom change from pre– to post–rehabilitation and, thus, a better mental health status post–rehabilitation.

Overall, the average scores showed an improvement from pre–rehabilitation treatment to post-rehabilitation treatment with regard to symptoms of depression, symptoms of anxiety, and perceived stress across the control group and intervention groups (see Figure 1a–d). For perceived loneliness, however, a reduction in perception was found for intervention groups 1 and 2, but not for the control group.

The results of the ANCOVA showed significant between–group differences with regard to the decrease in symptoms in the outcome domains from pre–rehabilitation to post–rehabilitation. Hence, significant differences were found for symptoms of depression (F (2638) = 4.50, *p* < 0.05, η_p_^2^ = 0.02) and symptoms of anxiety (F (2638) = 4.19, *p* < 0.05, η_p_^2^ = 0.02); however, significant differences were not found for perceived stress (F (2638) = 2.38, *p* = 0.09, η_p_^2^ = 0.01) and perceived loneliness (F (2638) = 2.39, *p* = 0.09, η_p_^2^ = 0.01).

Bonferroni’s post hoc test indicated a significant difference between the KG and IG2 (M_diff_ = −0.74, *p* = 0.40) and a significant difference between IG1 and IG2 (M_diff_ = −0.55, *p* = 0.02) for symptoms of depression. With regard to symptoms of anxiety, Bonferroni’s post hoc test indicated a significant difference between IG1 and IG2 (M_diff_ = −0.58, *p* = 0.02).

Looking at mean scores in Table 1, this effect is highlighted by the results for depression and anxiety of the IG2 group reporting a significantly decreased symptom intensity post-rehabilitation. In addition, the average symptoms for perceived stress and perceived loneliness post-rehabilitation were lowest in intervention group 2. These results suggest an improved mental health, especially for intervention group 2.

### 3.4. Effect Sizes

Effect sizes were estimated for the outcome domains of symptoms of depression, anxiety, and perceived stress between the measurement time points (pre– and post–rehabilitation) and for group comparison purposes post-rehabilitation. From pre– to post–rehabilitation, Cohen’s d values indicated a medium effect for overall symptoms of depression (0.69) and symptoms of anxiety (0.69). From pre– to post–rehabilitation, the effect size of Cohen’s d values, taking the intervention and control group into consideration, for depression was significantly larger in the IG2 group (1.27) compared to the IG1 group (0.66) and to the control group (0.31). For symptoms of anxiety, Cohen’s d values were significantly larger in the IG2 group (1.08) than in the IG1 group (0.66).

### 3.5. Association between Reaching Rehabilitation Goals Post-Rehabilitation and Satisfaction with Rehabilitation Post-Rehabilitation

To evaluate the association between reaching rehabilitation goals post-rehabilitation and satisfaction with rehabilitation treatment, a stepwise hierarchical regression analysis was performed controlling for age, gender, and intervention group with rehabilitation goals as predictors (Table 2). 

Results underlined that patients who indicated to have achieved the following rehabilitation goals were also more satisfied with the overall rehabilitation treatment process: reduction of psychological symptoms (b = 0.20, *p* < 0.01), improvement of physiological status (b = 0.14, *p* < 0.01), relaxation and resting (b = 0.14, *p* < 0.01), improvement of coping with stress and management of stress (b = 0.12, *p* < 0.05), and improvement of one’s own confidence (b = 0.11, *p* < 0.05). However, results showed a non-significant difference between treatment groups associated with the estimation of reaching rehabilitation goals post-rehabilitation (F (16, 1314) = 1.524, *p* = 0.08, η_p_^2^ = 0.02). In addition, no significant differences regarding satisfaction with rehabilitation treatment were found between the intervention groups (F (2682) = 0.02, *p* = 0.98, η_p_^2^ = 0.01; see Table 2).

### 3.6. Association between Perceived Communication and Satisfaction with Rehabilitation Post-Rehabilitation

To evaluate the association between perceived effectiveness of communication and satisfaction with rehabilitation treatment post-rehabilitation, a hierarchical stepwise regression was performed controlling for age, gender, and the intervention groups with perceived effectiveness of communication as a predictor (see Table 3).

Results showed that rehabilitation patients who indicated higher effectiveness of communication were also more satisfied with their rehabilitation treatment on the following communication dimensions: early enough discussion on treatment steps and plans (b = 0.22, *p* < 0.01), taking worries and fears seriously (b = 0.25, *p* < 0.01), and provision of sufficient information (b = 0.12, *p* < 0.05). The covariates of intervention group (b = 0.01, *p* = 0.91), age (b = 0.01, *p* = 0.98), and gender (b = 0.02, *p* = 0.47) were not significantly associated with the relationship between perceived communication and satisfaction with treatment (Table 3).

## 4. Discussion

The present study assessed the decrease in symptoms of depression, anxiety, perceived stress, and loneliness from pre– to post–rehabilitation by also evaluating the effectiveness of different digital trainings offered to medical, psychosomatic rehabilitation patients with regard to symptom reduction in the aforementioned mental health outcome domains. The digital trainings were implemented for medical, psychosomatic rehabilitants in preparation for their rehabilitation stay. Thus, they were implemented under the conditions of the German rehabilitation system, which is characterized by interdisciplinary care in sectoral organization, and applications by insured persons [46]. 

Furthermore, this study also assessed the association between perceived effectiveness with communication and satisfaction with the rehabilitation process as well as with having achieved rehabilitation goals. In general, the digital trainings seemed to be a suitable part of the rehabilitation if participants achieved their rehabilitation goals.

### 4.1. Reduction in Mental Health-Related Symptoms and the Effectiveness of Different Digital Trainings

Previous research has already indicated that the rehabilitation process is able to support symptom reduction in patients form a medical, psychosomatic clinic [47,48,49]. This is in line with our results, highlighting that symptoms of depression, anxiety, stress, and perceived loneliness decreased significantly from pre– to post–rehabilitation, irrespective of the intervention or control group. These findings provide insight that offering psychotherapy in addition to regular interventions, such as occupational therapy, relaxation, and physiotherapy, supports the symptom reduction of not only ICD-10 diagnoses, such as depression and anxiety, but also of symptoms associated with ICD-10 diagnoses, such as perceived stress and loneliness. The results were significant irrespective of patients’ age or gender. 

However, when examining the interaction effect between symptom change over time and intervention group, significant differences were only found with regard to symptoms of depression and anxiety. It may be postulated that reducing symptoms of stress and loneliness is not the central goal of the German medical, psychosomatic rehabilitation system and treatment process, as stress and loneliness are not considered as ICD-10 diagnoses. The overall treatment process is formulated on that basis of the theoretical biopsychosocial model of the International Classification of Functioning, Disability, and Health (ICF) and with consideration of the ICD-10 diagnosis. Hence, complaints are, thus, translated by diagnostic tests into diagnoses, which are necessary and a prerequisite for the treatment process [50,51]. Consequently, symptoms of stress and loneliness may not be specifically targeted by the different digital interventions offered in addition to the regular treatment process. It may be suggested that, because loneliness and stress are central sustaining factors for depression and anxiety, the digital interventions should be adapted to also reduced these symptoms respectively.

With regard to the effectiveness of different digital trainings offered during rehabilitation, results have highlighted that, for patients receiving different combinations of digital trainings (i.e., training on rehabilitation goals and training on legal rights for (severely) disabled), an average symptom reduction was found for depression, anxiety, and perceived stress. We found the same for participants who were part of the control group as well. However, perceived loneliness did not decrease for patients as part of the control group. Hence, the present results would suggest a beneficial effect of the rehabilitation setting especially with regard to depression, anxiety, and perceived stress. These findings are consistent with previous findings [47,48,49].

This was especially pronounced when comparing the patients allocated to the control group with patients from intervention group 1 (digital rehabilitation goals) as well as patients from intervention group 1 with participants from intervention group 2 (digital rehabilitation goals, group therapy on depression, and digital training on legal rights for (severely) disabled) concerning symptom reduction in depression. With regard to symptom reduction in anxiety, this effect was significant when comparing intervention group 1 with intervention group 2. Hence, intervention group 2 was shown to be significantly superior with regard to symptom reduction concerning depression and anxiety. Considering symptom reduction of perceived stress and perceived loneliness, intervention group 2 was shown to be on average superior to either intervention group 1 or the control group. These findings indicate that the interdisciplinary and multidisciplinary medical, psychosomatic rehabilitation program as a whole had a positive impact on mental health status, a finding that adds to previous research [51,52]. However, the long-lasting effects of the interdisciplinary rehabilitation program were not analyzed as part of this study and will need to be considered when assessing stabilization of mental health, return to work (RTW), and social participation after rehabilitation. Hence, further research is necessary to evaluate the mentioned research questions and to validate the results of the present study.

Despite the promising results highlighted by the present study, it needs to be stressed that digital trainings as part of the psychosomatic rehabilitation process need to constantly be tailored, evaluated, adapted and modified to the needs of the patients, to current treatment guidelines, as well as to the newest scientific developments to ensure an effective care and treatment program as well as overall patient safety.

### 4.2. Interpretation of Effect Sizes

So far, effect size benchmarks have only been postulated for regular face-to-face treatments but not for digital trainings as part of a medical, psychosomatic treatment process. Previous literature has defined effect size benchmarks for psychologically based treatment programs from pre– to post–treatment stay [47,53]. As part of the study, the authors suggested average effect sizes across different measurement domains, among others for depression to be at 0.35, which has been recommended to be used for the assessment of treatment programs. In the present study, the pre– to post–treatment effect sizes (Cohen’s d) for outcome variables (i.e., symptoms of depression (0.69) and symptoms of anxiety (0.69)) were revealed to be of medium effect size. Effect sizes across all symptom outcome domains were largest in the IG2 group, i.e., Cohen’s d for depression was 1.27 and 1.08 for anxiety. Therefore, our results are in line with the proposed effect size benchmarks by Fenton and Morley [53] and Liebherz and Rabung [47]. Hence, future research should focus on recommending effect size benchmarks for digital trainings in a medical, psychosomatic rehabilitation setting.

### 4.3. Rehabilitation Goals

Results stressed that patients who perceived greater satisfaction with rehabilitation goals (i.e., reduction of psychological symptoms, improvement of physiological status, relaxation and resting, improved stress coping capabilities, and improvements in own confidence and self-esteem) also displayed greater satisfaction with the overall rehabilitation treatment process. Additionally, patients who were more satisfied with their treatment also perceived greater satisfaction with communication (i.e., early discussions on treatment steps and plans, taking patients’ worries and fears seriously, and providing sufficient information). However, no significant difference was found with regard to the intervention groups. These results are in line with previous literature on the potentials of telemedicine generally and especially in times of crisis like the COVID-19 pandemic [6].

### 4.4. Limitations and Suggestions for Future Research

One of the main limitations of the current study is that we had no indication of the mental health status of psychosomatic rehabilitation patients before the outbreak of the COVID-19 pandemic. Hence, we cannot be certain whether the COVID-19 pandemic was associated with an aggravated symptom increase with respect to symptoms of depression, anxiety, stress, and loneliness, as shown by previous literature [49,54]. A further limitation that needs to be discussed is that participation in the digital training on rehabilitation goals and in legal rights for (severely) disabled was on a voluntary basis. Hence, it may have occurred that patients who were especially motivated to work on their symptoms and benefited from the treatment procedures offered during the rehabilitation stay, also participated in more digital interventions. Therefore, future studies should also consider motivational factors. 

In addition, this study did not consider possible confounding correlations of physiological symptoms (i.e., disabilities, chronic pain, cancer-related illness, or a potential COVID-19 infection) with mental health. Another limitation that needs to be considered is that the digital trainings offered before and during the rehabilitation stay so far have not been validated or standardized, but rather align with the German regulations for rehabilitation treatments and were developed based on experiences by the rehabilitation clinics. Hence, with regard to future research, a standardized manual, such as the Curriculum Hannover for aftercare [55], should be developed to effectively integrate standardized and evaluated digital trainings into the rehabilitation treatment process. In Germany, a cultural particularity of rehabilitation is that rehabilitation is mostly done in in-patient settings and has to be applied for with pension insurance funds by insured persons. The main goal is social and work participation. In other cultural contexts, the digital trainings may not be applicable without adaptation due to the rather unique nature of the German rehabilitation system that aims to bring the UN Disability Rights Convention into practice. Due to the historical development, the rehabilitation system in Germany is a complex system to ensure social security against diseases, unemployment, age, and disability. 

In addition, another limitation concerns the Cronbach’s alpha value of rehabilitation goals prior to the rehabilitation stay that has been found to be relatively low at 0.65 [56] compared to post-rehabilitation (α = 0.89). It may be postulated that the items presented to the patients may be, on the one hand, heterogeneous in their nature, since individual rehabilitation goals differ; on the other hand, they may not be as relevant to the sample population pre–rehabilitation compared to post–rehabilitation. Patients may have been unsure of what to expect and how to work on their undertaken goals. Hence, this may also be an indicator about the need to provide more effective communication prior to the rehabilitation stay to patients about expectations and goals as well as on how to set and work on rehabilitation goals.

As countries are becoming more culturally diverse, further research should also be considered to replicate and validate the current findings in countries with different rehabilitation systems and with patients from different cultural backgrounds. With regard to the different cultural expression of emotions and acknowledgements of psychological disorders, as well as acceptance of psychotherapy as a form of treatment, the question remains whether different healthcare systems and psychosomatic rehabilitation treatments (i.e., delivered at a higher proportion in a digital mode or even solely digital) would result in a similar outcome arises.

## 5. Conclusions

The findings of this study suggest that medical, psychosomatic rehabilitation is effective in reducing symptoms related to mental health disorders. By providing new technologies, i.e., digital elements as part of the healthcare services and the treatment process, symptoms of depression, anxiety, perceived stress, and perceived loneliness could be reduced post-rehabilitation. This was especially the case with the multidisciplinary and interdisciplinary rehabilitation treatment program, i.e., a treatment program including digital rehabilitation goals, digital group therapy for depression, and a digital training on legal rights for the (severely) disabled. They were shown to be especially effective with regard to symptom reduction of depression and anxiety, which are the central goals of the medical, psychosomatic rehabilitation process. Furthermore, greater satisfaction with the rehabilitation process was associated with the perception of rehabilitation goals as well as with greater satisfaction with communication between patients and healthcare professionals.

## Figures and Tables

**Table 1:** Descriptive statistics (estimated marginal means (M), and standard deviations (SD)) for treatment outcomes for all treatment groups from pre–rehabilitation to post–rehabilitation (*n* = 705).

Measure	Group	Pre–Treatment	Post–Treatment
		Mean (SD)	Mean (SD)
Symptoms of Depression			
	CG	3.48 (1.87)	2.59 (1.70)
	IG1	3.43 (1.63)	2.33 (1.68)
	IG2	3.67 (1.51)	2.01 (1.08)
Symptoms of Anxiety			
	CG	3.73 (1.74)	2.61 (1.69)
	IG1	3.56 (1.63)	2.47 (1.66)
	IG2	3.83 (1.56)	2.24 (1.36)
Perceived Stress			
	CG	9.84 (2.83)	7.00 (3.35)
	IG1	9.35 (2.31)	6.78 (3.27)
	IG2	9.68 (2.04)	6.45 (2.96)
Perceived Loneliness			
	CG	4.51 (1.74)	4.54 (1.66)
	IG1	4.29 (1.63)	4.17 (1.66)
	IG2	4.56 (2.00)	4.13 (1.64)

Note. CG = control group (n = 55; no digital intervention except regular rehabilitation treatment), IG1 = intervention group 1 (n = 570, in addition to regular rehabilitation treatment participation in digital rehabilitation goals), IG2 = intervention group 2 (n = 80, in addition to regular rehabilitation treatment participation in digital rehabilitation goals, digital group therapy on depression, and on legal rights for (severely) disabled).

**Table 2:** Step-wise hierarchical regression results predicting satisfaction with rehabilitation post-rehabilitation by reaching rehabilitation goals in *n* = 663 rehabilitation patients.

	Model 1		Model 2		Model 3		Model 4		Model 5		Model 6	
	ß	*p*	ß	*p*	ß	*p*	ß	*p*	ß	*p*	ß	*p*
Age	0.07	0.09	0.05	0.12	0.05	0.17	0.04	0.25	0.03	0.37	0.02	0.46
Gender	0.05	0.23	0.06	0.06	0.07	0.05	0.07	0.03	0.07	0.03	0.07	0.03
Intervention Group	−0.01	0.84	−0.03	0.32	−0.02	0.55	−0.02	0.51	−0.02	0.61	−0.03	0.45
Reduction of psychological symptoms	–	–	0.50	<0.01	0.38	<0.01	0.27	<0.01	0.23	<0.01	0.20	<0.01
Improvement of physiological status	–	–	–	–	0.22	<0.01	0.18	<0.01	0.15	<0.01	0.14	<0.01
Improvement of coping with stress and management of stress	–	–	–	–	–	–	0.19	<0.01	0.16	<0.01	0.12	0.01
Relaxation and resting	–	–	–	–	–	–	–	–	0.15	<0.01	0.14	<0.01
Improvement of one’s own confidence	–	–	–	–	–	–	–	–	–	–	0.11	0.01
R^2^		0.01		0.26		0.29		0.31		0.32		0.33

Note. ß-values are represented as standardized coefficients. Age was categorized into below 29 years of age, 30–39 years of age, 40–49 years of age, 50–59 years of age, and above 60 years of age. Gender was categorized into male and female.

**Table 3:** Stepwise hierarchical regression results: predicting satisfaction with rehabilitation post-rehabilitation by perceived effectiveness of communication in *n* = 641 rehabilitation patients.

	Model 1		Model 2		Model 3		Model 4	
	ß	*p*	ß	*p*	ß	*p*	ß	*p*
Age	0.05	0.24	0.02	0.50	0.01	0.78	0.01	0.98
Gender	0.04	0.29	0.01	0.76	0.02	0.55	0.02	0.47
Intervention Group	−0.01	0.89	0.01	0.89	0.01	0.78	0.01	0.91
Taking worries and fears seriously	–	–	0.48	<0.01	0.29	<0.01	0.25	<0.01
Early enough discussion on treatment steps and plans	–	–	–	–	0.28	<0.01	0.22	<0.01
Sufficient provision of information	–	–	–	–	–	–	0.12	0.03
R^2^		0.01		0.24		0.28		0.29

Note. ß-values are represented as standardized coefficients. Age was categorized into below 29 years of age, 30–39 years of age, 40–49 years of age, 50–59 years of age, and above 60 years of age. Gender was categorized into male and female.

## Data Availability

The data presented in this study are available on request from the corresponding author. The data are not publicly available due to confidential patient data being used.
